# Genomic and transcriptomic characterization of skull base chordoma

**DOI:** 10.18632/oncotarget.13616

**Published:** 2016-11-25

**Authors:** Jason K. Sa, In-Hee Lee, Sang Duk Hong, Doo-Sik Kong, Do-Hyun Nam

**Affiliations:** ^1^ Graduate School of Health Science & Technology, Samsung Advanced Institute for Health Science & Technology (SAIHST), Sungkyunkwan University, Seoul, Korea; ^2^ Samsung Biomedical Research Institute, Samsung Medical Center, Seoul, Korea; ^3^ Institute for Refractory Cancer Research, Samsung Medical Center, Seoul, Korea; ^4^ Department of Otorhinolaryngology, Samsung Medical Center, Sungkyunkwan University School of Medicine, Seoul, Korea; ^5^ Department of Neurosurgery, Samsung Medical Center, Sungkyunkwan University School of Medicine, Seoul, Korea

**Keywords:** skull base chordoma, genomic characterization, transcriptomic characterization, gene fusion, T gene

## Abstract

Skull base chordoma is a primary rare malignant bone-origin tumor showing relatively slow growth pattern and locally destructive lesions, which can only be characterized by histologic components. There is no available prognostic or therapeutic biomarker to predict clinical outcome or treatment response and the molecular mechanisms underlying chordoma development still remain unexplored. Therefore, we sought out to identify novel somatic variations that are associated with chordoma progression and potentially employed as therapeutic targets. Thirteen skull base chordomas were subjected for whole-exome and/or whole-transcriptome sequencing. In process, we have identified chromosomal aberration in 1p, 7, 10, 13 and 17q, high frequency of functional germline SNP of the *T* gene, rs2305089 (*P* = 0.0038) and several recurrent alterations including *MUC4*, *NBPF1*, *NPIPB15* mutations and novel gene fusion of *SAMD5*-*SASH1* for the first time in skull base chordoma.

## INTRODUCTION

Chordoma is a rare malignant tumor arising from notochordal remnant, mainly involving the skull base, sacrococcygeal area and vertebral bodies[1]. Skull base chordoma only accounts for 1~4% of all primary bone sarcomas[2] and is clinically characterized by relatively slow growth pattern and locally destructive lesions, infiltrating into adjacent critical anatomical structures. Although skull base chordoma is pathologically benign, this clinically malignant tumor is widely resistant to conventional radio- and chemotherapy [3], leaving surgical resection as the only mainstay of treatment option. However, complete tumor removal remains challenging due to its proximity of adjacent neurovascular complexity, thus leaving a subset of residual tumor cells, which in turn may persist to induce tumor relapse. Furthermore, there have been limited studies on genome-based research of skull base chordoma to suggest any alternative therapeutic option. Therefore, comprehensive integrated approach of transcriptome profiling and genome-wide analysis is essential in identifying promising molecular biomarker to provide a therapeutic solution for this rare yet aggressive disease.

Chordoma is known to express the transcription factor *T* (brachyury) and this *T* gene (6q27) is closely associated with pathogenesis[4]. Although few studies have revealed chromosomal aberration in sacral and skull base chordomas including frequent deletions of chromosome 9[5, 6] and 10[6], harboring *CDKN2A* and *PTEN*, respectively, and gain of chromosome 7[7], little is known about the nucleotide substitution, chromosomal and structural rearrangements to drive tumorigenesis. In the present study, we have characterized genomic and/or transcriptomic profiles across thirteen skull base chordomas including Copy Number Alterations (CNAs), Single Nucleotide Variations (SNVs), Insertions/Deletions (Indels), and structure rearrangements including exon deletions and gene fusions to identify novel and potentially important targets for prognosis, diagnosis, and therapeutics.

## RESULTS

### Whole-exome sequencing identifies genomic aberrations in skull base chordoma

**Figure 1:**
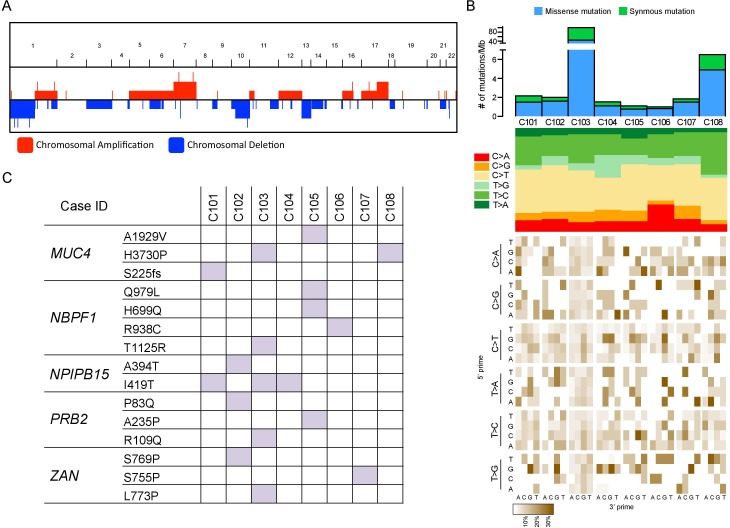
Genomic features of skull base chordoma **A.** Genome-wide copy number alteration including amplification/deletion in skull base chordomas. X-axis represents chromosome 1 to 22 and Y-axis represents the relative number of cases. **B.** Top panel shows number of mutations (substitutions and indels) per Mb (megabase) per sample. Middle panel notes mutation type, indicating mutation spectrum of each sample. Bottom panel represents mutation context, showing base substitution mutation spectra for each mutation found in the sample. Each of the 96 mutated trinucleotides is represented in a heatmap. The base corresponding to 5′ is shown on the vertical axis, and the 3′ base is on the horizontal axis. **C.** Summary of recurrent mutations that were identified in skull base chordomas. Mutations that were observed at least in more than three cases were selected.

To explore genome-wide alterations including CNAs, SNVs, and Indels, eight tumor specimens were subjected for whole-exome sequencing (Table 1). We observed several common chromosomal aberrations including loss of 1p, 10, and 13 and gain of 7 and 17q in our cohort (Figure 1a), consistent with the previous reports [8, 9]. Both chromosomal gain and loss of 7 and 10, respectively, are common genetic lesions, frequently observed in major malignant tumors including glioblastoma [10, 11], melenoma [12], and prostate cancer, [13] suggesting these alterations could be associated with tumor progression in chordoma as well.

**Table 1:** Summary of clinical features of skull base chordoma

No	Age	Gender	Subtype	Location	Extent of resection	Primary/recurrence	Postopertive Radiation	Survival
1	56	M	Chondroid	Clivus	GTR	Recurrence	Y	alive
2	67	M	Chondroid	Clivus	GTR	Primary	N	alive
3	49	M	Chondroid	Clivus	STR	Primary	N	alive
4	63	F	Chondroid	Clivus	GTR	Primary	N	alive
5	62	M	Typical	Clivus	GTR	Primary	Y	alive
6	64	F	Typical	Clivus	GTR	Primary	N	alive
7	72	M	Typical	Clivus	GTR	Recurrence	N	alive
8	60	M	Chondroid	Clivus	GTR	Recurrence	Y	alive
9	58	M		Clivus	GTR	Primary	ND	alive
10	48	M		Clivus	STR	Recurrence	ND	alive

GTR, gross total resection of tumor; STR, subtotal resection of tumor; ND, not determined

Mutational analyses revealed average mutation rate of 2.1 mut/Mb for non-hypermutated samples and most of the base substitution transitions were from C-to-T, T-to-C, and C-to-A. Although no consistent mutation sequence context was observed, four cases were characterized by prominence of C-to-T substitutions at NpCpG trinucleotides (Figure 1b), a mutational signature that is often detected in various cancer classes such as lymphoma, glioma, kidney chromophobe and etc. [14]. It has been previously suggested that such event is related to the elevated rate of spontaneous deamination of 5-methyl-cytosine, which results in C-to-T transitions [15]. In addition, we found several recurrent mutations including *MUC4*, *NBPF1*, and *NPIPB15* that were concurrently observed in more than three cases (Figure 1c). Both *MUC4* and *NBPF1* were previously described to be associated with promotion of tumor growth in pancreatic adenocarcinoma [16] and tumor invasion suppression in cervical cancer [17]. Interestingly, albeit no formerly known association with tumor, *NPIPB15* I419T point mutation was unanimously detected in three cases (Figure 1c), suggesting its variation could be a common event in Chordoma progression such as *IDH1* in glioma [18, 19] and acute myeloid leukemia (AML) [20, 21]. Further validation in a larger cohort could strength this observation.

### Structural rearrangements and transcriptomic profiles of skull base chordoma

**Figure 2:**
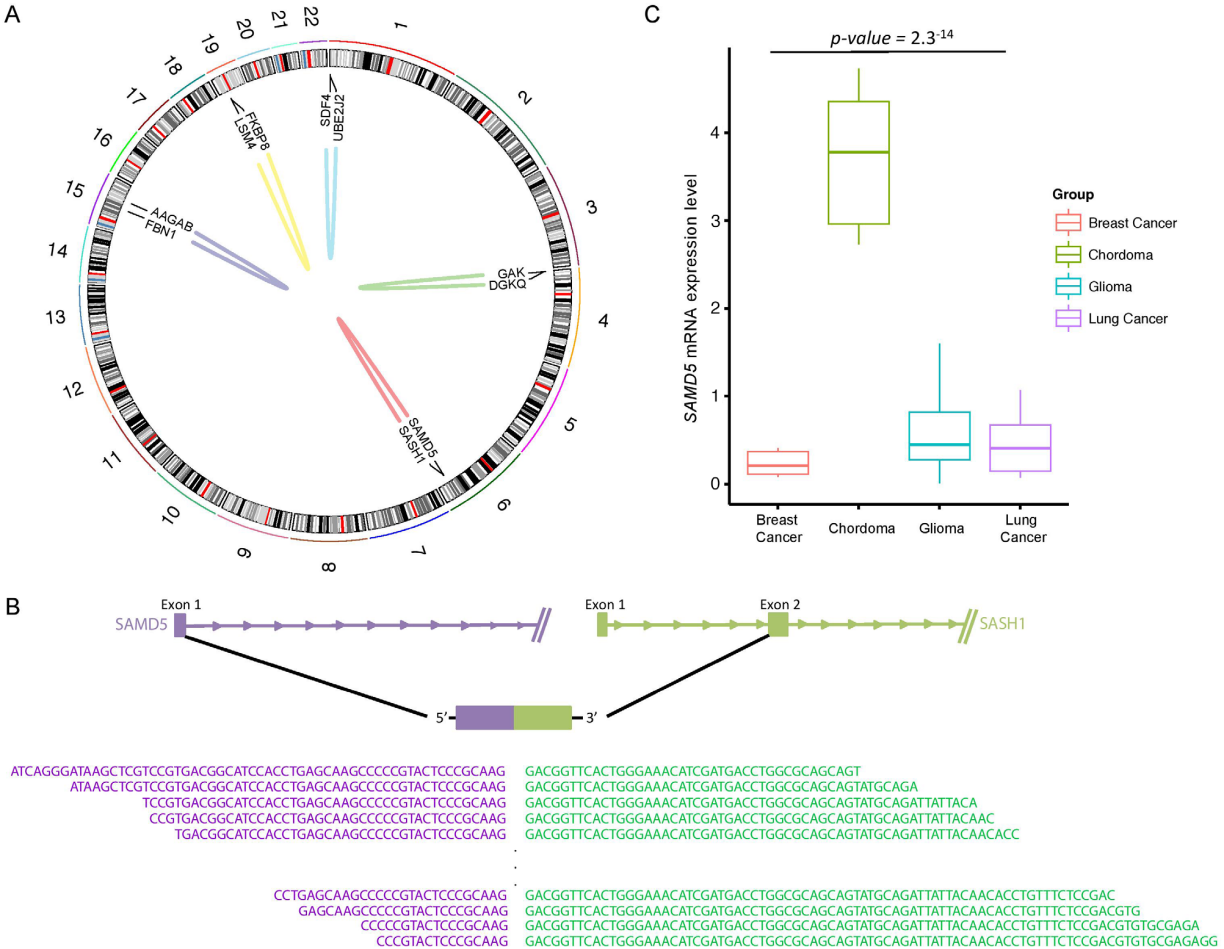
Structural rearrangements and transcript variants in skull base chordoma **A.** A Circos plot displaying intra-chromosomal structural rearrangements. Outer ring represents chromosome 1 to 22 and fusion gene annotations are marked in the center map. Each line in the center map represents a single structural variant to the site of origin for both genes. **B.** A schematic of spliced transcripts of the fusion gene, *SAMD5*-*SASH1*. Bottom sequences are the actual reads that map onto the splicing junction. **C.** Relative mRNA expression of *SAMD5* in breast cancer adenocarcinoma, skull base chordoma, glioma and lung cancer. mRNA expression level has been log2 transformed.

To explore gene expression patterns and transcriptomic structural rearrangements, we performed RNA sequencing (RNA-seq) across five skull base chordoma specimens. In total, we identified five distinctive intrachromosomal translocations including a recurrent in-frame fusion of *SAMD5* with *SASH1* in four individual cases (Figure 2a). Both *SAMD5* and *SASH1* are located on human chromosome 6q24 and they are 772kb apart. Our analysis has detected a breakpoint coordinate to chromosome 6 (147,830,523 for *SAMD5* and 148,711,270 for *SASH1*), falling within exon 1 and 2 of *SAMD5* and *SASH1*, respectively (Figure 2b). The fusion transcripts were further validated by RT-PCR (Supplementary Figure 1a). Furthermore, as gene fusion often results in elevated gene expression level, we assessed transcriptome level of *SAMD5* in skull base chordoma with different tumor types with available RNAseq data. We found that chordoma group has significantly higher *SAMD5* expression level compared to the other major cancers, further validating its fusion event (Figure 2c). In addition, we verified that the average mRNA expression level of *SAMD5* in the fusion harboring samples was significantly higher compared to the non-fusion sample (Supplementary Figure 1b).

Gene fusion has been identified as the major driver mutation and recent studies have revealed its functional role in tumor initiation and progression [22, 23]. However, since gene fusions associated with recurrent amplicons could represent passenger events, as they are the by-products of chromosomal amplification [24], we further verified copy number alteration of *SAMD5* and *SASH1* and found no abnormal aberration in samples with the fusion. Taken together, these data suggest that the fusion transcript of *SAMD5*-*SASH1* could potentially be involved in skull base chordoma development and employed as a therapeutic and prognostic marker.

### Alteration in the *T* gene in skull base chordoma

**Figure 3:**
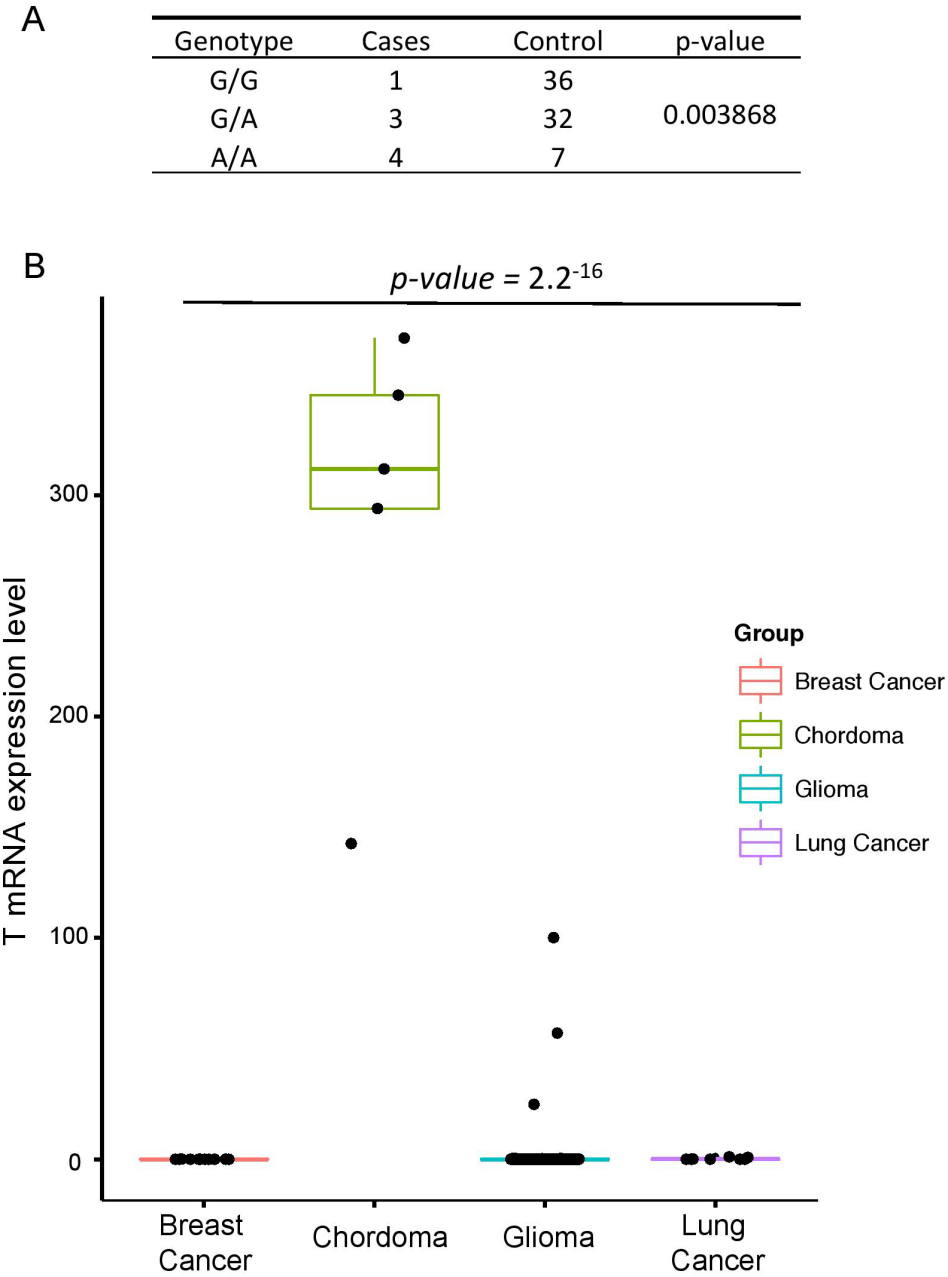
*T* gene alteration in skull base chordoma **A.** Genotype frequencies of the *T*, brachyury, gene Gly177Asp single-nucleotide polymorphism in skull base chordoma and control group. *P* values were calculated using Pearson''s χ-squared test. **B.** Relative mRNA expression of *T* gene in breast cancer adenocarcinoma, skull base chordoma, glioma and lung cancer.

The brachyury gene, also known as the *T* gene, expression has been widely regarded as the diagnostic marker for Chordoma [1]. Previous studies have shown that the presence of common functional SNP, rs2305089 (Gly177Asp) in the *T* gene is strongly associated with chordoma risk in European population [4]. They have identified that the frequency of the A allele at rs2305089 is 86% in individuals from European ancestry and poses higher risk of developing chordoma. However, association between the *T* gene and chordoma development in Korean population remains unknown. Therefore, we assessed the distribution of the rs2305089 SNP in Korean population, patients who were diagnosed with either skull-base chordoma or glioma as the control population. Notably, genotype frequencies of the brachyury rs2305089 SNP differed significantly between the chordoma group and the control group as 7 out of 8 patients with skull base chordoma (~88%) harbored the SNP at both heterozygous (G/A) and homozygous (A/A) genotypes (three and four cases for G/A and A/A, respectively), whereas only 39 out of 75 patients (~52%) in the control group harbored either G/A or A/A genotypes (32 and 7 cases, respectively) (Figure 3a). In addition, previous studies have shown that the absence of *T* gene SNP was associated with poor prognosis in chordoma[25]; however, we found no significant differences in terms of survival rate (data not shown).

Furthermore, transcriptome profile has revealed elevated expression level of the *T* gene in chordoma patients compared to those from different cancer background including glioma, lung cancer and breast cancer adenocarcinoma (Figure 3b). Interestingly, increased expression level of the *T* gene was associated with the presence of SNP only in the skull base chordoma. Overall, our results highlight the significance of *T* alteration in chordoma progression in Korean population.

## DISCUSSION

Cancer is a complex disease with diverse molecular basis and genetic aberrations, leading to its malignant transformation. In an attempt to comprehend functional importance of genetic alterations that drive tumor initiation and evolution, comprehensive genomic characterization of various tumors has been studied extensively, most notably lead by the nation-wide efforts of The Cancer Genome Atlas (TCGA)[11, 26–28]. These studies have identified profound oncogenic pathways including TP53, Phosphoinositide 3-kinase (PI3K), and Receptor Tyrosine Kinases (RTKs) that are frequently involved in tumor propagation. While these studies have revealed crucial insights into tumor architecture and biology, there still remain other cancer classes that are yet to be investigated.

Although, much studies involving sacral chordoma have shown profound results[29–32], genomic determinants for skull base chordoma progression remain elusive, urging a need for further examination. Towards this goal, we have characterized genomic and transcriptomic profiles of skull base chordomas. Our results have revealed chromosomal abnormality in 1p, 7, 10, 13, and 17 regions and recurrent somatic variants including *MUC4*, *NBPF1*, *NPIPB15* single nucleotide variations and *SAMD5*-*SASH1* gene fusion. These alterations could potentially be involved in skull base chordoma initiation and development; therefore further functional validation of these variations could discover molecular mechanism behind its malignant transformation. Furthermore, we show that the occurrence of this rare tumor is strongly associated with the presence of the germline functional SNP rs2305089 in the *T* gene. Specifically, we estimate that the frequency of the A allele at rs2305089 is 88%, similar to the previous studies on European ancestry. Overall, genomic profiling of skull base chordoma will be of great value as it may provide crucial insights into the tumorigenic pathways of skull base chordoma progression.

## MATERIALS AND METHODS

### Clinical manifestations

Between 2009 and 2016, 10 patients with skull base chordoma underwent endoscopic transclival resections. There were 2 female and 8 male patients. The mean patient age at the time of diagnosis was 59.9 years (range 48-72 years). Of the total patients, 6 patients presented with newly diagnosed primary clival tumors, and 4 patients had recurrent tumors (previously treated) (Table 1). The median follow-up period was 83 months (range 8–132 months). Histologically, there were 5 chondroid chordomas and 5 typical (classical) chordomas. Gross-total tumor removal was performed in 8 cases and subtotal removal as resection of > 90% of the tumor, was achieved in 2 cases.

### Chordoma specimens

Following informed consent in accordance with the appropriate Institutional Review Boards, chordoma specimens were obtained from patients undergoing surgery. For genomic analysis, tumor specimens that were diagnosed by the pathologists were snap-frozen and preserved in liquid nitrogen. Genomic DNA and mRNA were extracted using the DNeasy kit and the RNeasy kit (Qiagen), respectively.

### Whole-exome sequencing

### Raw data

Agilent SureSelect kit was used for capturing exonic DNA fragments. Illumina HiSeq2000 was used for sequencing and generated 2 × 101 bp paired-end reads.

### Somatic mutation

The sequenced reads in FASTQ files were aligned to the human genome assembly (hg19) using Burrows-Wheeler Aligner version 0.6.2. The initial alignment BAM files were subjected to conventional preprocessing before mutation calling including sorting, removing duplicated reads, realigning reads around potential indels, and recalibrating base quality scores using SAMtools, Picard version 1.73 and Genome Analysis ToolKit (GATK version 2.5.2.) We used MuTect (version 1.1.4) and SomaticIndelDetector (GATK version 2.2) to make high-confidence predictions on somatic mutations from the neoplastic and non-neoplastic tissue pairs. Variant Effect Predictor (VEP) was used to annotate somatic mutations.

### Copy number

ngCGH python package was used to generate aCGH-like data from WES. Matching normal samples were used as the reference for calculating copy number variations in tumors. In cases in which patient-matched normal samples were not available, we created a “pseudo-normal” profile as the reference (Reference Kim Cancer Cell 2015). Segmentation and copy number calculation of each gene were performed.

### RNA sequencing

Trimmed sequenced reads of 30 nucleotides (nt) were mapped on hg19 using GSNAP to exclude any mismatch, indels, or splicing. SAMtools sorted the aligned SAM files and bedTools was used to summarize into BED files. DEGseq was used to calculate RPKM (Reads Per Kilobase of transcript per Million reads) values. For gene fusion analysis, subsequent filters were applied to the results to identify the paired-end reads that satisfy the following criteria: 1) each end aligns to different genes, 2) either end aligns to the intron where a break-point is suspected to be found based on the exon expression profile from the per-nucleotide coverage analysis. The identified paired-ends were mapped on hg19 to reveal fusion points.

### Quantitative real-time PCR

RNAs were extracted (Qiagen) and their complementary DNAs were synthesized (Invitrogen) per manufacturer's instructions. Duplicate reactions were performed for each set of primers and the relative amounts of target transcripts were normalized to the number of human beta-actin transcripts. The relative quantification of target gene expression was performed with the comparative cycle threshold (CT) method. Real-time PCR was performed using the following primer.

## SUPPLEMENTARY FIGURE


