# Supplementary material for: Genomic and transcriptomic characterization of skull base chordoma
Source: Oncotarget. 2016 Nov 25;8(1):1321–8. doi: 10.18632/oncotarget.13616 (PMC5352057; doi:10.18632/oncotarget.13616)
Supplement: Supplementary file 1 [file oncotarget-08-1321-s001.pdf]

Genomic and transcriptomic characterization of skull base chordoma

SUPPLEMENTARY FIGURE

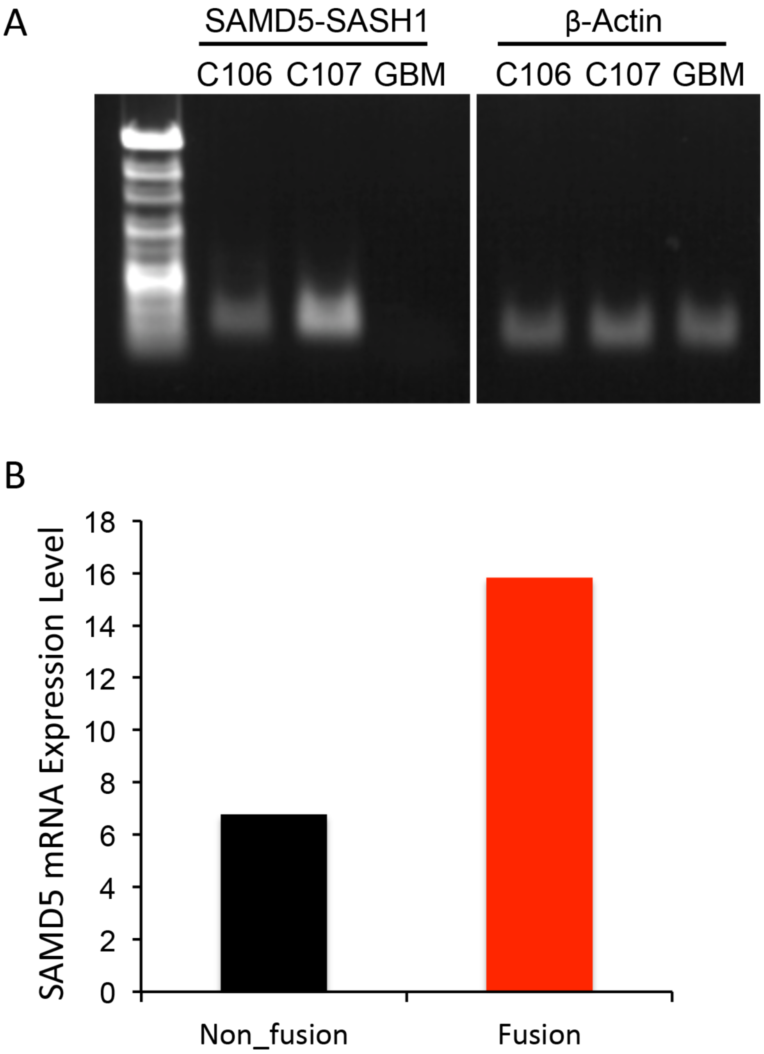

Supplementary Figure S1: qRT-PCR of SAMD5-SASH1 fusion and SAMD5 mRNA expression level. A. *SAMD5-SASH1*-specific PCR from cDNA derived from skull base chordoma harboring *SAMD5-SASHI1* fusion. GBM was used as a control for comparison. **B.** Relative mRNA expression of *SAMD5* gene in *SAMD5-SASH1* fusion harboring samples vs. non-harboring sample.
